# Developing and validating a multivariable prediction model which predicts progression of intermediate to late age-related macular degeneration—the PINNACLE trial protocol

**DOI:** 10.1038/s41433-022-02097-0

**Published:** 2022-05-25

**Authors:** Janice Sutton, Martin J. Menten, Sophie Riedl, Hrvoje Bogunović, Oliver Leingang, Philipp Anders, Ahmed M. Hagag, Sebastian Waldstein, Amber Wilson, Angela J. Cree, Ghislaine Traber, Lars G. Fritsche, Hendrik Scholl, Daniel Rueckert, Ursula Schmidt-Erfurth, Sobha Sivaprasad, Toby Prevost, Andrew Lotery

**Affiliations:** 1 Clinical and Experimental Sciences, Faculty of Medicine, University of Southampton, Southampton, UK; 2 BioMedIA, Imperial College London, London, UK; 3 Institute for AI and Informatics in Medicine, Klinikum rechts der Isar, Technical University Munich, Munich, Germany; 4 Christian Doppler Laboratory for Ophthalmic Image Analysis, Medical University of Vienna, Vienna, Austria; 5 Institute of Molecular and Clinical Ophthalmology, Basel, Switzerland; 6 NIHR Moorfields Biomedical Research Centre, Moorfields Eye Hospital NHS Foundation Trust, London, UK; 7 Department of Ophthalmology, Landesklinikum Mistelbach-Gänserndorf, Mistelbach, Austria; 8 Department of Biostatistics, University of Michigan School of Public Health, Ann Arbor, MI USA; 9 Nightingale-Saunders Clinical Trials and Epidemiology Unit, King’s Clinical Trials Unit, King’s College London, London, UK

**Keywords:** Education, Outcomes research

## Abstract

**Aims:**

Age-related macular degeneration (AMD) is characterised by a progressive loss of central vision. Intermediate AMD is a risk factor for progression to advanced stages categorised as geographic atrophy (GA) and neovascular AMD. However, rates of progression to advanced stages vary between individuals. Recent advances in imaging and computing technologies have enabled deep phenotyping of intermediate AMD. The aim of this project is to utilise machine learning (ML) and advanced statistical modelling as an innovative approach to discover novel features and accurately quantify markers of pathological retinal ageing that can individualise progression to advanced AMD.

**Methods:**

The PINNACLE study consists of both retrospective and prospective parts. In the retrospective part, more than 400,000 optical coherent tomography (OCT) images collected from four University Teaching Hospitals and the UK Biobank Population Study are being pooled, centrally stored and pre-processed. With this large dataset featuring eyes with AMD at various stages and healthy controls, we aim to identify imaging biomarkers for disease progression for intermediate AMD via supervised and unsupervised ML. The prospective study part will firstly characterise the progression of intermediate AMD in patients followed between one and three years; secondly, it will validate the utility of biomarkers identified in the retrospective cohort as predictors of progression towards late AMD. Patients aged 55–90 years old with intermediate AMD in at least one eye will be recruited across multiple sites in UK, Austria and Switzerland for visual function tests, multimodal retinal imaging and genotyping. Imaging will be repeated every four months to identify early focal signs of deterioration on spectral-domain optical coherence tomography (OCT) by human graders. A focal event triggers more frequent follow-up with visual function and imaging tests. The primary outcome is the sensitivity and specificity of the OCT imaging biomarkers. Secondary outcomes include sensitivity and specificity of novel multimodal imaging characteristics at predicting disease progression, ROC curves, time from development of imaging change to development of these endpoints, structure-function correlations, structure-genotype correlation and predictive risk models.

**Conclusions:**

This is one of the first studies in intermediate AMD to combine both ML, retrospective and prospective AMD patient data with the goal of identifying biomarkers of progression and to report the natural history of progression of intermediate AMD with multimodal retinal imaging.

## Introduction

AMD is the commonest cause of blindness in the elderly. By 2020, 200 million people are expected to be affected with AMD, increasing to nearly 300 million by 2040 [1]. It is a complex, heritable and heterogeneous disease that affects the macula, the central retina that is responsible for detailed central vision. The pathogenesis of AMD is unclear despite intensive research. Drusen are an early clinical feature of the disease and are visualised as yellow deposits predominantly located throughout the macula. However, drusen are also a feature of normal aging adding to the complexity of disease classification. Currently, the severity of AMD is classified into three stages. Early and intermediate AMD are classified based on the size and morphology of drusen and pigmentary changes at the macula while late AMD is characterised by geographic atrophy (GA) and abnormal blood vessel growth. These classification systems do not adequately capture the wide range of phenotypic variation seen in AMD eg the wide range of ‘intermediate AMD’; the lumping together of geographic atrophy (GA) and macular neovascularization (MNV) in ‘advanced AMD’; the absence of acquired vitelliform lesion as a form of AMD etc.

In addition, the inter-individual progression rates of intermediate AMD to advanced forms are extremely variable. For example, currently there are no specific anatomical markers for disease progression that can reliably predict individual risk of conversion to late AMD [2, 3]. Furthermore, AMD classifications are still based on colour fundus photographs despite significant advancement in multimodal imaging of the retina. For example, quantification of drusen volume on OCT is a more reliable quantitative marker than a large drusen identified on colour fundus photographs [4]. However, drusen load is dynamic over time [4]. Therefore, although change in numbers, areas and volume of drusen may provide us with a composite score on overall disease progression, ML may be a better option to identify the fate of each drusen in an eye to understand focal conversion to late AMD. Late AMD may present as geographic atrophy (GA) with atrophy of photoreceptors and retinal pigment epithelium (RPE) in the macula or choroidal neovascularization (CNV) where choroidal blood vessels migrate into the retina and it remains unknown why, when and which markers/events may precede or predict each form of late AMD. If it was possible to predict progression to late AMD this may have ethical implications if no treatments were available to treat it e.g. there are currently no available treatments for GA. Patients would have to be counselled appropriately in this scenario. However, the ability to predict who might progress faster could be advantageous for stratification of patients into clinical trials and in the longer term to allow better personalised medicine if novel treatments are developed as we hope.

Recent advances in imaging technologies have enabled identification of a wide spectrum of potential imaging biomarkers of AMD such as reticular pseudodrusen (subretinal deposits) that develop and progress independently of drusen [5]. Optical coherence tomography (OCT) also indicates that there are 38 different types of drusen that may co-exist in a patient and their significance in AMD progression is unknown [6]. Degeneration of neurosensory layers of the retina was recently shown to play a major role in the prediction of progression to late AMD [7].

Successes in ML during recent years have enabled new applications in medicine and healthcare. In medical imaging, machine learning has been successfully employed for image analysis and interpretation, including [8, 9] the discovery of biomarkers for disease progression and providing decision support for clinicians regarding diagnostic and therapeutic management [10, 11]. In order to exploit these advances to gain better understanding of AMD and provide a personalised risk prediction model for AMD, there are several technical challenges that need to be addressed.

A key challenge is the detection and quantification of changes to retinal structures, which are known to be associated with AMD. This task can be addressed using deep learning techniques which allow highly accurate automated segmentation of anatomical structures. Subsequently, biomarkers such as the volume, density or shape of the segmented structures can be derived and analysed. Using convolutional neural networks, our group has pioneered automated segmentation of intra-/subretinal fluid, hyperreflective foci, neurosensory layers, posterior hyaloid interfaces and the retinal vasculature in AMD eyes [12]. These supervised machine learning approaches have been highly successful as long as there is sufficient training data that has been annotated by human expert observers. Additionally, a hypothesis is needed which structures and associated biomarkers may be of interest.

Another promising avenue is unsupervised biomarker discovery, which can be used to obtain an unbiased, clinically meaningful summary of OCT data. The detected image features can be either directly used for diagnostic or prognostic tasks or presented to clinical experts so that they can formulate hypotheses for new biomarkers. We have recently pioneered this field by defining clinically and genetically distinct disease endophenotypes in nvAMD [13] as well as by discovering so far unknown anomalies in OCT data using a novel technology called generative adversarial networks [14].

The large, natural variability of structure and function across different subjects and populations makes it difficult to determine or learn biomarkers predictive of disease progression. One way of dealing with this heterogeneity is the use of computational models to provide a common reference space. Such spatio-temporal atlases do not only account for anatomic variations and idiosyncrasies of each individual subject, but also offer a powerful framework which facilitates comparison of anatomy over time, between subjects, between groups of subjects and across sites. They have been widely used to capture spatio-temporal processes in the brain, such as neurodevelopment and the progression of Alzheimer’s disease [15–18]. Therefore, a spatio-temporal atlas seeks to define normal anatomy at different ages. It can then be used to extract rules from a machine learning perspective to detect abnormal structural patterns that develop overtime which may be related to abnormal outcomes. In the context of ophthalmology, it could be used to detect structural biomarkers predicting progression to late AMD from OCT retinal images that have been used to create such an atlas of normal retinal ageing.

Finally, decision support tools should support clinical diagnosis and prognosis predictions by making the process more efficient, reliable and objective. Making personalised diagnostic predictions from imaging data is a challenge that can be addressed by combining disease progression models and spatio-temporal models in a statistical model that is learnt from data. There have been initial attempts to do so using machine learning and colour fundus photographs in ophthalmology [15, 19]. There is considerably more information available by utilising optical coherent tomograms (OCTs) of the retina. We have previously used spatio-temporal input features derived from OCT and demonstrated that this approach confers substantial advantages in predicting the future course of retinal disease in terms of prediction of drusen fate on a spatially resolved individual drusen level, progression of AMD on a per-eye level, as well as visual outcomes and treatment intervals in therapy of AMD [17, 20, 21].

## Objectives

Following on from the preliminary work described above the objectives of the PINNACLE study are:

1. To characterise focal and global changes of pathological ageing of the retina to accurately distinguish between health and early pathological changes of AMD disease.
2. To identify and quantify pathognomonic markers and sequence of events preceding cell death and progression to late AMD.
3. To improve accuracy of classification of AMD by building traditional and machine learning models, integrating data from multiple large sources.
4. To create a personalised risk prediction model for progression to late AMD which is accurate and clinically relevant.

## Subjects and methods

### Study design

PINNACLE is a multicentre, non-interventional prospective predictive modelling study with a large-scale retrospective data study running alongside as described below.

### Retrospective study

For the retrospective part of the project, we collected OCT scans of patients 50 years of age or above from routine clinical practice from four university hospital sites: University Hospital Southampton, Moorfields Eye Hospital, Medical University of Vienna, and University Hospital Basel. To increase the rate of healthy subjects, we also integrated OCT data of the UK Biobank biomedical database. In total, this pooled dataset contains more than 400,000 OCT images from almost 100,000 subjects (Table 1).

**Table 1:** Sources of OCT scans of AMD patients and healthy subjects that are being pooled in the scope of the retrospective study.

Dataset source	# OCT	# subjects	Comments
University Hospital Southampton	57,875	4251	Primarily acquired with Topcon OCT
Moorfields Eye Hospital	154,371	3956	Acquired with Topcon OCT
Medical University of Vienna	40,626	1649	Acquired with Cirrus OCT
University Hospital Basel	15,976	618	Acquired with Spectralis OCT
UK Biobank	175,869	85,721	Mostly healthy eyes (Topcon OCT)
Total	444,717	96,195	

These images are centrally pseudonymised, standardised, preprocessed and stored. With the help of the associated clinical records, the data is filtered and curated into OCT scans of healthy eyes (negative control), and scans of AMD patients, which will be further stratified into eyes with early AMD (at risk population) and eyes with late AMD (positive control). For many patients, OCT scans taken at multiple timepoints are available that display the longitudinal progression of the disease and healthy aging. A subset of the longitudinal dataset will be labelled by retinal experts. For this purpose, fellow eyes of eyes under treatment with frequent follow-up will be manually graded for conversion from normal retina to AMD based on the presence of soft drusen. These treated eyes may have had treatment for a variety of reasons eg late AMD or retinal vein occlusion. For conversion to advanced neovascular AMD grading will be based on the presence of intraretinal or subretinal fluid, pigment epithelial detachment and subretinal hyperreflective material. Grading of conversion to advanced atrophic AMD will be based on the presence of complete retinal pigment epithelium and outer retinal atrophy (cRORA) [22]. Additionally, experts may identify and label relevant retinal structures in a subset of images.

Using this large retrospective dataset of AMD patients and healthy subjects, we aim to automatically quantify and track the development of known indicators (OCT biomarkers) of retinal aging and AMD. We will train and employ image segmentation algorithms to localise and segment anatomical structures, such as neurosensory retinal layers, vasculature, drusen, hyperreflective foci, intra-/subretinal fluid, pigment epithelial detachment, subretinal hyperreflective material and early signs of geographic atrophy [23].

We can only curate a limited amount of training data for these algorithms due to the costly and time-consuming nature of manual data annotation by clinical experts. In order to still benefit from the large number of OCT images, we will explore the use of self-supervised pretraining. Self-supervised pretraining teaches neural networks to extract semantic information without the need for labelled training data [23, 24]. It may facilitate the use of deep learning with small labelled training datasets or boost its peak performance compared to conventional one-stage training.

Beyond the use of machine learning to extract known retinal features, we aim to use unsupervised machine learning to identify and define novel imaging biomarkers that predict disease progression, characterise AMD and classify subjects into different populations (i.e. fast progressors versus slow progressors). We will construct a spatio-temporal atlas that enables us to compare the retinal anatomy across time for an individual subject, between subjects and between different groups of subjects [25, 26]. It is expected that such atlas and associated representations will facilitate identification of structural changes in the outer retina over time that may shed light on the biological process by which these changes convert to late AMD.

Informed by these findings, we will design and train AI-based prognostic models capable of: (i) predicting the onset of early/intermediate AMD from scans of healthy retina, and (ii) predicting the onset of late neovascular and/or late atrophic AMD from scans of retina with intermediate AMD. The first model would provide a risk estimate (time to event) of AMD onset, providing an insight into AMD risk factors, while the second model will provide a risk estimate (time to event) of advanced AMD onset, providing an insight into risk factors for AMD progression, which will be crucial for patient management and to start a timely treatment for both neovascular and atrophic AMD stages.

The utility of all biomarkers and derived prognostic models will be validated using a separate partition of the large retrospective dataset and generated labels. The multicentre data and different OCT scanners used allow us to assess their generalisability. Ultimately, the identified biomarkers and models will be benchmarked in the prospective study, where an exploratory outcome will be how accurately they predict progression to focal event development.

### Prospective study

Eligible patients who give written informed consent will be followed by OCT and other imaging modalities every 4 months to detect the earliest focal sites of disease progression. OCT, OCT-A and fundus autofluorescence imaging will be acquired using the Heidelberg spectralis retinal angiograph (Heidelberg Engineering Gmbh, Heidelberg, Germany). Adaptive optics imaging will be acquired via an RTX1 adaptive optics retinal camera (Imagine Eyes, Orsay, France). Microperimetry will be acquired via a Maia microperimeter (Centervue S.p.A, Padova, Italy). The trial schedule is shown in Table 2. Focal areas of change will trigger a targeted follow-up schedule to investigate the events at that focal area of change. Recruitment will take place across 12 different hospital sites: four main hospital sites Vienna, Basel, Southampton and Moorfields and 8 UK hospital referral sites feeding patients with focal events into the two UK main hospital sites (Southampton and Moorfields) for in-depth imaging. The 8 UK hospital referral sites are Bristol Eye Hospital, Guys Hospital, John Radcliffe Hospital, Princess Alexandra Hospital, Salisbury District Hospital, St Mary’s Hospital Isle of Wight, and St Mary’s Hospital London.

**Table 2:** List of Measures Taken at Visits.

Procedures	Visit number (Month)										Focal event visits	
	1 (0)	2 (4)	3 (8)	4 (12)	5 (16)	6 (20)	7 (24)	8 (28)	9 (32)	10 (36)	1	2
Demographics	☑☑											
Medical history	☑☑											
Concomitant medications	☑☑											
Physical examination	☑☑											
Blood sample for DNA analysis	☑☑											
Ocular examination	☑☑			☑☑			☑☑			☑☑		
BCVA	☑☑			☑☑			☑☑			☑☑		
LLVA	☑☑			☑☑			☑☑			☑☑		
Colour fundus photography	☑☑			☑☑			☑☑			☑☑		
OCT	☑☑	☑☑	☑☑	☑☑	☑☑	☑☑	☑☑	☑☑	☑☑	☑☑	☑☑	☑☑
OCT-A	☑	☑	☑	☑	☑	☑	☑	☑	☑	☑	☑☑	☑☑
FAF	☑			☑			☑			☑	☑☑	☑☑
Adaptive optics	☑			☑			☑			☑		
Microperimetry	☑			☑			☑			☑	☑☑	☑☑
Adaptive optics at focal event visit											☑☑	☑☑

☑: Main site only.

☑☑: Main site & referral site.

*BCVA* best-corrected visual acuity, *LLVA* low luminance visual acuity, *FAF* fundus autofluorescence, *OCT* optical coherence tomography, *OCT-A* optical coherence tomography angiography.

All UK sites are secondary NHS Trusts with two further main sites located at University teaching hospitals in Basel and Vienna. In the UK, this study has been approved by the East Midlands - Leicester Central Research Ethics Committee (ref. 19/EM/0163); in Austria, by the ethics committee of the Medical University of Vienna; and in Switzerland by a national central research ethics committee, SWISSETHICS. The principles of Good Clinical Practice will be adhered to throughout in accordance with the Declaration of Helsinki.

**Fig. 1:**
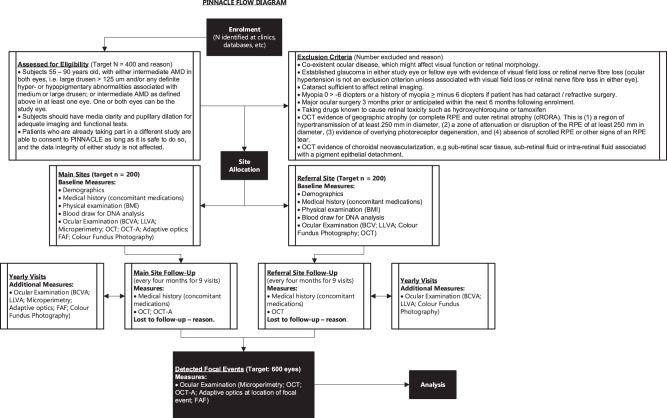
Pinnacle flow diagram.

Patients are informed about the planned number of visits and duration of their participation in the study as part of their informed consent. The trial schema showing the recruitment pathway, follow-up schedule and assessments of patients is shown in Fig. 1. Recruitment started in October 2019 and will end in June 2022. Follow-up will be a minimum of 2 years and maximum of 3 years.

## Outcomes

### Primary outcome

Relating to the fourth study objective, the primary endpoint for an individual participant is the occurrence of a focal event, defined as development of early-onset neurosensory atrophy or macular neovascularisation during the study. The primary analysis will be a Cox model of the time from baseline to either censored follow-up or to a primary endpoint. This analysis will provide the primary outcome of the study which is the identification of those particular candidate predictors (biomarkers and established risk factors) that remain important in the model, after backwards elimination, in jointly best predicting progression to a focal event. Key supportive outcomes, related to the understanding of this primary outcome, comprise: (a) the particular weighted combination, or relative importance, of these remaining important predictors; (b) corresponding with the model, an implied overall performance measure of the area under the ROC curve will also provide a range of estimates of sensitivity and specificity to demonstrate personalised predicted risk for individuals of progression to focal events; (c) the univariate performance of every candidate predictor will be transparently presented using sensitivity, specificity, and ROC curve where the biomarker is not a binary variable.

### Secondary outcomes

Sensitivity and specificity of novel imaging characteristics e.g. fundus autofluorescence (FAF), adaptive optics flood illumination ophthalmoscopy (AO-FIO), OCT-A at predicting disease progression; ROC curves; time from development of imaging change to development of these end-points; functional outcomes: BCVA, LLVA, microperimetry; structure-function correlation; structure-genotype correlation; predictive risk models.

### Exploratory outcomes

Within the prospective cohort of patients, in addition to the primary outcome we will record subsequent focal events and other AMD-related events which may occur any number of times during the follow-up period, in order to perform exploratory analysis of the progression to late AMD development.

## Study population for prospective study

### Inclusion criteria

Subjects 55–90 years old, with either intermediate AMD (as defined by ref. [27]) in both eyes, i.e. large drusen > 125 μm and/or any definite hyper- or hypopigmentary abnormalities associated with medium or large drusen; or intermediate AMD as defined above in one eye (study eye) and advanced AMD (geographic atrophy or choroidal neovascularization secondary to AMD) in the other eye.

Subjects should have media clarity and pupillary dilation for adequate imaging and functional tests.

Patients who are already taking part in a different study are able to consent to PINNACLE as long as it is safe to do so, and the data integrity of either study is not affected.

Exclusion criteria:

1. Co-existent ocular disease, which might affect visual function or retinal morphology.
2. Established glaucoma in either study eye or fellow eye with evidence of visual field loss or retinal nerve fibre loss (ocular hypertension is not an exclusion criterion unless associated with visual field loss or retinal nerve fibre loss in either eye).
3. Cataract sufficient to affect retinal imaging.
4. Myopia ≥ -6 dioptres or a history of myopia ≥ -6 dioptres if patient has had cataract/refractive surgery.
5. Major ocular surgery 3 months prior or anticipated within the next 6 months following enrolment.
6. Taking drugs known to cause retinal toxicity such as hydroxychloroquine or tamoxifen.
7. In the study eye: OCT evidence of geographic atrophy (or complete RPE and outer retinal atrophy (cRORA)). This is (1) a region of hypertransmission of at least 250 μm in diameter, (2) a zone of attenuation or disruption of the RPE of at least 250 μm in diameter, (3) evidence of overlying photoreceptor degeneration, and (4) absence of scrolled RPE or other signs of an RPE tear.
8. In the study eye: OCT evidence of choroidal neovascularization e.g. sub-retinal scar tissue, sub-retinal fluid or intra-retinal fluid associated with a pigment epithelial detachment.

### Harms and safety reporting

All serious adverse events (SAEs) occurring during the prospective study observed by the investigator or reported by the participant, will be noted on the CRF and reported to sponsor within 24 h of discovery.

The following information will be recorded: description, date of onset and end date, severity, assessment of relatedness to study, other suspect device and action taken. Follow-up information should be provided as necessary.

SAEs considered related to the study as judged by a medically qualified investigator or the sponsor will be followed until resolution or the event is considered stable. All related SAEs that result in a participant’s withdrawal from the study or are present at the end of the study, should be followed up until a satisfactory resolution occurs.

It will be left to the investigator’s clinical judgement whether or not a SAE is of sufficient severity to require the participant’s removal from the study. A participant may also voluntarily withdraw from study due to what he or she perceives as an intolerable SAE. If either of these occurs, the participant must undergo an end of study assessment and be given appropriate care under medical supervision until symptoms cease or the condition becomes stable.

The severity of events will be assessed on the following scale: 1 = mild, 2 = moderate, 3 = severe.

In addition to the reporting above, the CI shall submit an Annual Report to Leicester Central Research Ethics Committee who approved the study, which lists all SAEs that have occurred during the preceding 12 months.

### Recruitment

Patients will be recruited from participating hospital eye clinics. In addition, we will encourage community optometrists and patient charities to refer patients for participation.

### Consent

The participant must personally sign and date the latest approved version of the informed consent form before any study-specific procedures are performed.

Written versions of the participant information and Informed consent will be presented to the participants detailing no less than the exact nature of the study; the implications and constraints of the protocol; the known side effects and any risks involved in taking part. It will be clearly stated that the participant is free to withdraw from the study at any time for any reason without prejudice to future care, and with no obligation to give the reason for withdrawal.

The participant will be allowed as much time as wished to consider the information, and the opportunity to question members of the study team, their GP or other independent parties to decide whether they will participate in the study. Written Informed Consent will then be obtained by means of participant dated signature and dated signature of the person who presented and obtained the informed consent. The person who obtained the consent must be suitably qualified and experienced and have been authorised to do so by the Principal Investigator as detailed on the Delegation of Authority and Signature log for the study. The original signed form will be retained at the study site within the Trial Master File (TMF) or Investigator Site File (ISF). A copy of the signed Informed Consent will be given to participants and a copy retained in the participant medical notes.

### Prospective cohort analysis

We will conduct a prospective non-interventional study including 400 intermediate AMD patients (with 600 untreated intermediate AMD eyes, including both unilateral (AREDS IV) and bilateral (≥AREDS II)) over a minimum of 2 years to specifically investigate the morphological sequence of events preceding the conversion towards late AMD. All patients will be followed by OCT imaging every 4 months to detect the earliest focal sites of disease progression. As soon as focal areas of change (see below) are observed by the VRC, a targeted follow-up schedule will be triggered to investigate the events at that area of change in a targeted manner. 200 patients (main cohort) will undergo dense retinal phenotyping at 10 visits as per Fig. 1. Medical and smoking history, genotype and body mass index will also be included in the analysis. As well as structural tests, functional tests will be performed at baseline and yearly during the study using both microperimetry to identify focal changes and low luminance visual acuity to assess global changes. In case of focal progression, specific focal event microperimetry grids, which are tailored to the sites of progression, will be applied at all subsequent visits in order to functionally evaluate the morphological changes.

To increase sample size but make the study feasible an additional 200 patients at UK referral sites will undergo 4 monthly OCT and be referred to Southampton / Moorfields for dense phenotyping only if a focal event is detected by OCT (Fig. 1).

Signs of focal progression (as determined by the VRC) are defined as:

1. focal drusen regression or
2. occurrence of novel nascent GA [28] or
3. new-onset PED, SRHM or intra- or subretinal fluid.

If signs of incipient focal progression (defined above) are observed, patients will undergo an intermediate visit within 2 weeks of the site being notified by the VRC and a second 4 weeks after that for targeted imaging and functional testing at sites of incipient focal progression including:

1. AO camera to image photoreceptors
2. high-resolution SD-OCT and fundus autofluorescence (FAF*) to image RPE.
3. OCT angiography (OCT-A) to image choriocapillaris.
4. Microperimetry (specific focal event and standard grids) to evaluate retinal sensitivity.

### Genotyping

For the prospective cohort, we will extract DNA from blood samples that will be genotyped on a Infinium Global Screening Array at the Advanced Genomics Biomedical Research Core Facility at the University of Michigan. Genotype data will be subjected to various quality control filters, likely resulting in a set of over 500,000 polymorphic variants. Principal components will be generated and used to adjust for population stratification. Ancestry will be estimated by projecting all genotyped samples into the space of the principal components of the Human Genome Diversity Project reference panel using PLINK [29]. Pairwise kinship will be assessed and used to reduce the data to a maximal subset that contained no pairs of individuals with 3rd-or closer degree relationship [30]. Additional genotypes will be obtained through imputation with the latest reference panels of the Michigan Imputation Server [31, 32]. We will calculate the same effect size weighted polygenic risk score (PRS) to identify patients at high risk for AMD in the UK Biobank component of the retrospective study.

### Microperimetry

The participants’ study eye will be tested for retinal sensitivity and fixation stability with the MAIA microperimetry (CentreVue, Padova, Italy) device. This machine presents Goldmann III stimuli with a dynamic range of 0–36 dB and a maximum luminance of 318.3 cd/m^2^ on a background of 1.3 cd/m^2^. Included real-time fundus imaging and eye-tracking provide a secure correspondence of light stimulus and retinal location during the examination. Three distinct grids will be employed throughout the study, which all feature a 4–2 thresholding strategy. (1) The 12-point training grid will be presented to the participants at the beginning of every MAIA examination. (2) The 24-point PINNACLE standard grid, which is centred in the fovea and covers the central 10-degree diameter circle, will be performed yearly. (3) The PINNACLE focal event grid features arrays of five points, tailored to the retinal loci of focal events. In case of focal progression, focal event grids will be designed by the site investigator and will be applied at all subsequent study visits. At the end of each examination the metrics of mean sensitivity, Bivariate Contour Ellipse Area (BCEA) fixation indices (63 and 95%) and percentage of fixation points within 1° and 2° of the foveal centre (P1 and P2) together with the sensitivity at every testing point will be exported for analysis.

### Features to minimise bias

Selection bias will be avoided by ensuring that all participants are recruited using the same recruitment strategy preferably in a consecutive manner and this ensures a representative sample from study sites over the study period. There is no randomisation or therefore allocation bias in this study. All images will be pseudonymised and sent to the Vienna Reading Centre, an established digital image analysis platform based on US Food and Drug Administration (FDA) and European Medicines Agency (EMA) standards, which has significant experience of reporting images based on standard operating procedures. This will avoid performance and detection bias. Being based on baseline visit data, candidate predictors are expected to be free from missing data. Any data would seldom be assumed missing completely at random, though a range of planned methods including ones intended for this assumption when missing data is infrequent, and ones for missing at random assumptions will be considered [33, 34], with sensitivity analysis undertaken. Being analysed with the Cox model which accommodates censored outcomes, none of the primary outcome data from those who drop out before study completion will be excluded from the analysis. This assumes data is missing at random conditional on the included candidate predictors, and sensitivity analysis will be undertaken. Candidate predictors and analyses will be listed in a statistical analysis plan specified before access to the joining of predictors and outcomes.

### Sample size

With 400 patients (600 eyes) we expect 170 composite time-to-event focal events signifying progression to late AMD. Focal events include drusen regression, early-onset neurosensory atrophy or choroidal neovascularisation, assuming 20% dropout and different 50%/20% unilateral/bilateral event rates, and conservatively maintained at 20% at the point of development of an event in the study eye(s) based on drusen regression in 26% of drusen over 5-year follow-up [35], and an overall conversion rate of 32% in unilateral neovascular AMD eyes over 2 years [35], which is in line with other literature [36]. Lifetable analysis showed that 170 events would be expected with these rates and 20% 3-year dropout. This was robust in a subsequent calculation over altered variable follow-up durations and employing higher observed event rates. For event modelling projects there is no statistical null hypothesis and so sample size calculations are not based on power. Instead, a rule of thumb is used that ten events (or ten non-events, if smaller) are needed per baseline-visit candidate predictor accommodated in the full model. Therefore, the initial model development stage after 360-eye follow-up (102 events) will accommodate ten identified structural biomarkers and potential genetic and phenotypic predictors. The 240-eye model (68 events) validation stage includes: calibration and discrimination and provides an informative narrow 95% confidence interval for C-index widths of ~±0.08.

### Plan for statistical analysis

The cohort will be divided 3:2 into a Cox model development stage (240 patients; 360 eyes; 102 primary endpoints) allowing ten (102/10) candidate predictors [33] and a validation stage (160 patients; 240 eyes; 68 primary endpoints), to enable the identification of the important predictors (primary study outcome) and their relative importance, and other key supportive outcomes including the area under the ROC curve with sensitivity and specificity estimates. After full recruitment, the patients defining these stages will be identified. Stage 1 modelling will begin prior to full follow-up. Candidate predictors will include identified structural biomarkers and potential genetic and phenotypic predictors. The initial full model will be a comparator and simplified with backwards elimination [37]. Continuous predictors will be included as fractional polynomials [38]. Models will include a unilateral/bilateral main effect to improve transportability to samples with an alternative case-mix. Intra-patient between-eye correlation will be accounted for with marginal models, with ‘eye’ the unit of prediction. Validation in Stage 2 will include calibration of the performance of the Stage 1 model [39] and discrimination via the C-index with sensitivity, specificity and predictive values, and assessment of any over-fitting bias.

External transportability of the model will involve meta-analytic methods. Fast progressors and early conversion to AMD will be explored, and short-term changes in predictors.

We will explore comparability of model parameters and C-indices in unilateral and bilateral subgroups, and composite constituents. We will follow approaches for modelling time-to-event data [33] and for external validation. The statistical analysis plan will be updated during the course of the study, for example: to record the definition of those biomarkers which are prioritised to be candidate predictors in the initial full model; to alter the number of candidate predictors that may be prioritised for accommodation in the full Cox model depending on the number of observed events achieved; to record any established risk factors that need to be initially assessed as candidate predictors; to handle missing baseline data in candidate predictors and over time in outcomes with sensitivity analysis; to record alternative methods should assumptions not be met; to record any secondary or exploratory additional analyses. These changes will be recorded within the final version of the statistical analysis plan which will be approved by the joint Trial Steering and Data Monitoring Committee (TSDC) prior to the joint access to both events and candidate predictors by the time of the final data lock.

### Trial management and monitoring

The study is overseen by a TSDC who will ensure the overall study integrity by monitoring progress, investigating and reviewing reports from the trial management group (TMG). It will consist of an independent Chair, independent retinal Specialists, and patient representatives. As there is no intervention or blinding of results it will also take on the role of a Data Monitoring Committee and will monitor the trial data to ensure accuracy, patient safety and ethical conduct, and will monitor recruitment data, adverse events, emerging external evidence, sample characteristics, primary outcomes and make recommendations regarding any interim analysis. The TSDC will meet yearly for the first 3 years.

Study documents, site initiation and training and day-to-day running of the study and monitoring of sites is being managed by the Clinical Trial Project Manager. A trial management group (TMG) (Chief Investigator, co-applicants, trial manager and other key members of the group) oversee the development and operation of the study, monitor and maintain recruitment rates. The TMG participate in teleconferences fortnightly to discuss the ongoing progress and meet annually to discuss the study progress as a whole.

The Trial Executive Group (TEG), chaired by the Chief Investigator, will collate and lead planning of publications encompassing data from all Collaborators. It is anticipated that each paper will have a writing committee involving the Co-Investigators who will then form the listed authors for the paper. Consensus will be sought from the TEG on authors and authorship order with a majority vote of TEG if needed being final.

### Protocol amendments

Version 1.1 was used when recruitment started (28 October 2019) and version 3.2 is currently in use. The main changes have been to the minimum change of follow-up. This has changed from 3 years to a minimum of 1. This has been due to the disruption of set up and recruitment during the COVID-19 pandemic. We have also changed the definition of a focal event. This was due to the original definition being too sensitive and picking up more than the relevant events. Frequency of microperimetry tests was increased to provide additional data on the focal events. Visit windows were also introduced to aid flexibility for participants and study teams, and the taking of blood samples was changed to allow it in any of the first 4 visits.

## Discussion

Using the retrospective element of this study we will identify OCT biomarkers through ML, which predict progression of intermediate to late AMD. We will evaluate these OCT biomarkers in the prospective natural history study described here. We will also create a reference annotated dataset of OCT images that may be of use for future ML projects. Focal events as defined here will enable us to stratify the prospective cohort into fast and slow progressors. In addition, this study will extensively phenotype this cohort of patients with exploratory tools, i.e. adaptive optics as well as the functional measures of microperimetry and also OCT angiography and autofluorescence imaging to determine the most sensitive biomarkers of progression. The aim is that the results of this project will provide a comprehensive evaluation of imaging and genetic biomarkers associated with intermediate AMD progression. This may inform a better understanding of underlying cellular and molecular processes associated with AMD pathogenesis. It will also allow the generation of a probabilistic risk score to aid patient stratification and management on an individualised and population-based level.

The clinical set-up for this study was slower than anticipated due to the COVID-19 pandemic halting recruitment at many sites for months. UK patients from referral sites were also reluctant to travel to the two main UK sites, Southampton and Moorfields for detailed focal event phenotyping. An additional challenge was working with different ethics regulations in different countries. For example, in Switzerland we were not allowed to recruit patients over the age of 90 which meant for consistency across all sites we did not recruit patients at any site over the age of 90. Budget constraints meant we could not employ a full clinical trial centre for trial oversight which was also challenging in terms of trial management.

Features of national healthcare systems have to be considered: Most patients were recruited from tertiary hospital sites which meant that many patients already had advanced AMD in their second eye. Thus, our estimate that we will recruit two eyes from around 50% of patients appears optimistic. Many patients with intermediate AMD would not be followed up routinely in the hospital eye service in the UK so they welcomed being included in a study were they would be observed for evidence of advancing to late-stage AMD. Consequently, retention in the study has been higher than expected. Indeed, wet AMD has been diagnosed at an early asymptomatic stage in several patients allowing early treatment to be instituted.

## Summary

### What was known before

- Risk factors for AMD progression are known and AMD risk calculators available.
- However the sensitivity of risk calculators for AMD progression is poor.

### What this study adds

- This study will combine data from a large-scale retrospective analysis of OCT images with a prospective study of intermediate AMD patients.
- Using machine learning of high-resolution three-dimensional retinal images and deep phenotyping this study will aim to identify sensitive and objective markers of progression of intermediate AMD.
- Description of the temporal sequence of morphological changes in each layer of the retina, RPE and choroid, under simultaneous evaluation of functionality, during progression of AMD and ageing in the retina.
- Correlation of imaging markers with the genetic profile of each individual in the prospective study and endophenotypes described.
- Mechanistic insight should be gained on the aetiological factors that cause intermediate AMD to progress.
- The potential for future treatment to be individualised to each patient, allowing for treatment to be optimised.
- The potential for improved stratification of patients in clinical trials.

## Data Availability

Data from this project will be referenced and linked to in papers published from this project in peer-reviewed journals. This data will include 1. The atlas of central retinal ageing and patient specific risk model. This will be available via websites hosted by Imperial College London. 2. Relevant imaging datasets will be made available via the Vienna Reading Center where the data will be stored via application to this research consortium who will be custodians of the data. 3. Genotype data. This will be stored at the European Genome-phenome Archive (EGA) (https://www.ebi.ac.uk/training/online/course/genomics-introduction-ebiresources/ europeangenome-phenome-archive-ega). Small datasets derived from this work may also be held on ePrints Soton, for example those bits of data directly mentioned in a journal paper. These datasets can either be openly available or available on request (for example if ethics clearance is required). The University of Southampton can issue DOIs for these datasets and they will be held for a minimum of 10 years since the last access as per the University’s Research Data Policy.

## References

[1] Wong WL, Su X, Li X, Cheung CMG, Klein R, Cheng C-Y (2014). Global prevalence of age-related macular degeneration and disease burden projection for 2020 and 2040: a systematic review and meta-analysis. Lancet Glob Health.

[2] Schmitz-Valckenberg S, Sahel JA, Danis R, Fleckenstein M, Jaffe GJ, Wolf S (2016). Natural history of geographic atrophy progression secondary to age-related macular degeneration (Geographic Atrophy Progression Study). Ophthalmology.

[3] Sunness JS, Margalit E, Srikumaran D, Applegate CA, Tian Y, Perry D (2007). The long-term natural history of geographic atrophy from age-related macular degeneration: enlargement of atrophy and implications for interventional clinical trials. Ophthalmology.

[4] Yehoshua Z, Wang F, Rosenfeld PJ, Penha FM, Feuer WJ, Gregori G (2011). Natural history of drusen morphology in age-related macular degeneration using spectral domain optical coherence tomography. Ophthalmology.

[5] Sivaprasad S, Bird A, Nitiahpapand R, Nicholson L, Hykin P, Chatziralli I (2016). Perspectives on reticular pseudodrusen in age-related macular degeneration. Surv Ophthalmol.

[6] Khanifar AA, Koreishi AF, Izatt JA, Toth CA (2008). Drusen ultrastructure imaging with spectral domain optical coherence tomography in age-related macular degeneration. Ophthalmology.

[7] Schmidt-Erfurth U, Waldstein SM, Klimscha S, Sadeghipour A, Hu X, Gerendas BS (2018). Prediction of Individual Disease Conversion in Early AMD Using Artificial Intelligence. Investig Ophthalmol Vis Sci.

[8] Bengio Y, Courville A, Vincent P (2013). Representation learning: a review and new perspectives. IEEE Trans Pattern Anal Mach Intell.

[9] LeCun Y, Bengio Y, Hinton G (2015). Deep learning. Nature.

[10] Antila K, Lotjonen J, Thurfjell L, Laine J, Massimini M, Rueckert D (2013). The PredictAD project: development of novel biomarkers and analysis software for early diagnosis of the Alzheimer’s disease. Interface Focus.

[11] Soininen H, Mattila J, Koikkalainen J, van Gils M, Hviid Simonsen A, Waldemar G (2012). Software tool for improved prediction of Alzheimer’s disease. Neuro-degenerative Dis.

[12] Schlegl T, Waldstein SM, Vogl WD, Schmidt-Erfurth U, Langs G (2015). Predicting semantic descriptions from medical images with convolutional neural networks. Inf Process Med Imaging.

[13] Seeböck P, Waldstein SM, Donner R, Gerendas BS, Sadeghipour A, Osborne A (2017). Defining disease endophenotypes in neovascular AMD by unsupervised machine learning of large-scale OCT data. Investig Ophthalmol Vis Sci.

[14] Schlegl T, Seebock P, Waldstein SM, Schmidt-Erfurth U, Langs G. Unsupervised anomaly detection with generative adversarial networks to guide marker discovery. https://arxiv.org/abs/1703.05921. 10.1016/j.media.2019.01.010 30831356

[15] Bhuiyan A, Wong TY, Ting DSW, Govindaiah A, Souied EH, Smith RT (2020). Artificial intelligence to stratify severity of age-related macular degeneration (AMD) and predict risk of progression to late AMD. Transl Vis Sci Technol.

[16] Schmidt-Erfurth U, Waldstein SM, Klimscha S, Sadeghipour A, Hu X, Gerendas BS, et al. Prediction of Individual Disease Conversion in Early AMD Using Artificial Intelligence. Investig Ophthalmol Vis Sci. 2018;59:3199–208. 10.1167/iovs.18-24106 29971444

[17] Bogunovic H, Waldstein SM, Schlegl T, Langs G, Sadeghipour A, Liu X (2017). Prediction of Anti-VEGF treatment requirements in neovascular AMD using a machine learning approach. Invest Ophthalmol Vis Sci.

[18] Kuklisova-Murgasova M, Aljabar P, Srinivasan L, Counsell SJ, Doria V, Serag A (2011). A dynamic 4D probabilistic atlas of the developing brain. Neuroimage.

[19] Yan Q, Weeks DE, Xin H, Swaroop A, Chew EY, Huang H (2020). Deep-learning-based prediction of late age-related macular degeneration progression. Nat Mach Intell.

[20] Schmidt-Erfurth U, Bogunovic H, Klimscha S, Hu X, Schlegl T, Sadeghipour A (2017). Machine learning to predict the individual progression of AMD from imaging biomarkers. Investig Ophthalmol Vis Sci.

[21] Schmidt-Erfurth U, Bogunovic H, Sadeghipour A, Schlegl T, Langs G, Gerendas BS (2018). Machine learning to analyze the prognostic value of current imaging biomarkers in neovascular age-related macular degeneration. Ophthalmol Retin.

[22] Sadda SR, Guymer R, Holz FG, Schmitz-Valckenberg S, Curcio CA, Bird AC (2018). Consensus definition for atrophy associated with age-related macular degeneration on OCT: classification of atrophy report 3. Ophthalmology.

[23] Waldstein SM, Seeböck P, Donner R, Sadeghipour A, Bogunović H, Osborne A (2020). Unbiased identification of novel subclinical imaging biomarkers using unsupervised deep learning. Sci Rep.

[24] Azizi S. Big self-supervised models advance medical image classification. Proceedings of the IEEE/CVF International Conference on Computer Vision. Computer Vision Foundation New York, USA, 2021. p. 347–88.

[25] Vogl W-D, Bogunović H, Waldstein SM, Riedl S, Schmidt-Erfurth U (2021). Spatio-temporal alterations in retinal and choroidal layers in the progression of age-related macular degeneration (AMD) in optical coherence tomography. Sci Rep.

[26] Waldstein SM, Vogl WD, Bogunovic H, Sadeghipour A, Riedl S, Schmidt-Erfurth U (2020). Characterization of drusen and hyperreflective foci as biomarkers for disease progression in age-related macular degeneration using artificial intelligence in optical coherence tomography. JAMA Ophthalmol.

[27] Ferris FL, Wilkinson CP, Bird A, Chakravarthy U, Chew E, Csaky K (2013). Clinical classification of age-related macular degeneration. Ophthalmology.

[28] Wu Z, Luu CD, Ayton LN, Goh JK, Lucci LM, Hubbard WC (2014). Optical coherence tomography-defined changes preceding the development of drusen-associated atrophy in age-related macular degeneration. Ophthalmology.

[29] Li JZ, Absher DM, Tang H, Southwick AM, Casto AM, Ramachandran S (2008). Worldwide human relationships inferred from genome-wide patterns of variation. Science.

[30] Manichaikul A, Mychaleckyj JC, Rich SS, Daly K, Sale M, Chen WM (2010). Robust relationship inference in genome-wide association studies. Bioinformatics.

[31] Taliun D, Harris DN, Kessler MD, Carlson J, Szpiech ZA, Torres R (2021). Sequencing of 53,831 diverse genomes from the NHLBI TOPMed program. Nature.

[32] McCarthy S, Das S, Kretzschmar W, Delaneau O, Wood AR, Teumer A (2016). A reference panel of 64,976 haplotypes for genotype imputation. Nat Genet.

[33] Royston P, Altman DG (2013). External validation of a Cox prognostic model: principles and methods. BMC Med Res Methodol.

[34] Burton A, Altman DG (2004). Missing covariate data within cancer prognostic studies: a review of current reporting and proposed guidelines. Br J Cancer.

[35] Bogunovic H, Montuoro A, Baratsits M, Karantonis MG, Waldstein SM, Schlanitz F (2017). Machine learning of the progression of intermediate age-related macular degeneration based on OCT imaging. Investivg Ophthalmol Vis Sci.

[36] Ferris FL, Davis MD, Clemons TE, Lee LY, Chew EY, Lindblad AS (2005). A simplified severity scale for age-related macular degeneration: AREDS Report No. 18. Arch Ophthalmol.

[37] Royston P, Moons KGM, Altman DG, Vergouwe Y (2009). Prognosis and prognostic research: developing a prognostic model. BMJ.

[38] Sauerbrei W, Royston P (1999). Building multivariable prognostic and diagnostic models: transformation of the predictors by using fractional polynomials. J R Stat Soc: Ser A (Stat Soc).

[39] Altman DG, Vergouwe Y, Royston P, Moons KGM (2009). Prognosis and prognostic research: validating a prognostic model. BMJ.

