# Correction: Developing and validating a multivariable prediction model which predicts progression of intermediate to late age-related macular degeneration—the PINNACLE trial protocol

**DOI:** 10.1038/s41433-022-02131-1

**Published:** 2022-08-11

**Authors:** Janice Sutton, Martin J. Menten, Sophie Riedl, Hrvoje Bogunović, Oliver Leingang, Philipp Anders, Ahmed M. Hagag, Sebastian Waldstein, Amber Wilson, Angela J. Cree, Ghislaine Traber, Lars G. Fritsche, Hendrik Scholl, Daniel Rueckert, Ursula Schmidt-Erfurth, Sobha Sivaprasad, Toby Prevost, Andrew Lotery

**Affiliations:** 1 Clinical and Experimental Sciences, Faculty of Medicine, University of Southampton, Southampton, UK; 2 BioMedIA, Imperial College London, London, UK; 3 Institute for AI and Informatics in Medicine, Klinikum rechts der Isar, Technical University Munich, Munich, Germany; 4 Christian Doppler Laboratory for Ophthalmic Image Analysis, Medical University of Vienna, Vienna, Austria; 5 Institute of Molecular and Clinical Ophthalmology, Basel, Switzerland; 6 NIHR Moorfields Biomedical Research Centre, Moorfields Eye Hospital NHS Foundation Trust, London, UK; 7 Department of Ophthalmology, Landesklinikum Mistelbach-Gänserndorf, Mistelbach, Austria; 8 Department of Biostatistics, University of Michigan School of Public Health, Ann Arbor, MI USA; 9 Nightingale-Saunders Clinical Trials and Epidemiology Unit, King’s Clinical Trials Unit, King’s College London, London, UK

**Keywords:** Education, Outcomes research

Correction to: *Eye* 10.1038/s41433-022-02097-0, published online 25 May 2022

The original version of this article unfortunately contained an error in an author name. The correction is to the first name of Hrvoje Bogunovic, currently spelt ‘Hvroje’. The authors apologize for the error. The original article has been corrected.

